# The Potential Use of Cold-Pressed Coconut Oil By-Product as an Alternative Source in the Production of Plant-Based Drink and Plant-Based Low-Fat Ice Cream: The Rheological, Thermal, and Sensory Properties of Plant-Based Ice Cream

**DOI:** 10.3390/foods12030650

**Published:** 2023-02-02

**Authors:** Muhammed Zahid Kasapoglu, Osman Sagdic, Esra Avci, Zeynep Hazal Tekin-Cakmak, Salih Karasu, Rabia Sena Turker

**Affiliations:** 1 Department of Food Engineering, Faculty of Chemical and Metallurgical Engineering, Yildiz Technical University, Istanbul 34210, Turkey; 2 Bypro Functional Food and Biotechnology, Istanbul 34210, Turkey; 3 Department of Nutrition and Dietetics, Health Sciences Faculty, Istinye University, İstanbul 34010, Turkey; 4 Department of Pharmacology, Faculty of Pharmacy, Van Yuzuncu Yil University, Van 65080, Turkey

**Keywords:** plant-based ice cream, low-fat ice cream, coconut by-products, fat replacer

## Abstract

This study aimed to investigate the potential use of cold-pressed coconut oil by-products (COB) as a low-cost alternative source for plant-based drink and ice cream production. Firstly, a plant-based drink was produced from cold-pressed coconut oil by-products (COB drink) and compared with a commercial coconut drink. The fat, protein, and zeta potential values of coconut drink obtained from COB were higher than those of the commercial samples. In addition, the particle size value of the drink obtained from COB was found to be lower than that of the commercial drink. In the second stage, full-fat and low-fat plant-based ice cream samples using COB drink were produced and compared to control ice cream samples (produced by the commercial coconut drink) in terms of rheological, sensorial, and thermal properties. Rheological analysis showed that all plant-based ice cream samples indicated pseudoplastic, solid-like, and recoverable characteristics. Low-fat commercial control ice cream samples (C1) indicated the lowest K value (9.05 Pas^n^), whereas the low-fat plant-based ice cream sample produced by the COB drink (COB-3) exhibited the highest K value (17.69 Pas^n^). ΔH_f_ values of the plant-based ice cream samples varied from 144.70 J/g to 172.70 J/g. The low-fat COB ice cream stabilized with 3% COB and full-fat COB ice cream samples showed lower ΔH_f_ values than control ice cream samples, indicating that the COB ice cream showed desired thermal properties. The COB drink may be utilized in plant-based ice cream without altering sensory qualities, and low-fat ice cream could be manufactured in the same manner to attain full-fat ice cream quality characteristics. The results of this study demonstrated that COB can be successfully used as an inexpensive raw material source in the production of full-fat and reduced-fat vegetable-based ice cream.

## 1. Introduction

The milk of animal origin and dairy products, such as ice cream, are among the most consumed food groups worldwide. However, in recent years, the tendency towards animal products has decreased due to allergenic effects, lactose intolerance, veganism, and ethical reasons, and the tendency toward plant-based alternatives of these products has increased [1,2]. For this reason, the popularity of plant-based drinks has increased day by day worldwide. Plant-based sources, such as coconut, almonds, and hazelnuts, are used in the production of plant-based drinks. Coconut drinks are one of the most preferred plant-based beverages as they have nutritional benefits and a distinctive flavor [3]. The expense of these raw materials increases the cost of plant drink production. Therefore, alternative cheap sources are needed to reduce the cost of raw materials.

Cold-pressed oils are extracted mechanically using a screw press without the application of heat, solvents, or chemical treatments. The maximum temperature of cold-pressed oil production does not exceed 50 °C. Therefore, there is a growing trend in cold-pressed oil consumption due to their high bioactive compounds. In addition, after oil extraction, a by-product rich in protein and carbohydrates emerges [4]. Coconut oil consumption has increased in recent years around the world due to beneficial health and functional properties of the coconut oils. Coconut oils consist of 90% of saturated fatty acids and 62% of its total fatty acid composition is made up of medium chain triglyceride. The cold pressing technique is one of the most used dry extraction methods in the production of coconut oil [5]. After the production of cold-pressed coconut oil, a by-product with a very high fiber and protein content emerges. For this reason, cold-pressed coconut oil by-products are a good candidate as an inexpensive raw material for plant-based products. The utilization of cold-pressed coconut oil by-products in plant-based drinks can provide an important alternative to reduce the cost of plant-based drink products and can also gain a valuable by-product identity for the oil industry. 

Ice cream is a frozen and aerated oil-in-water (O/W) emulsion with a high amount of dairy or non-dairy fat (10–16%) combined with air bubbles, fat globules, and ice crystals [6]. The texture, mouthfeel, creaminess, and overall preferences of ice cream are enhanced by the interaction of fat with other ingredients. However, consumers prefer low-fat or low-calorie food products as a result of their association with a lower risk of coronary heart disease and obesity. Therefore, recent research focuses on the use of alternative ingredients and the right combination of ingredients that give the best texture when creating a new formulation for low-fat ice creams [7]. However, ice cream is a thermodynamically unstable product. For this reason, stability is ensured by using certain special additives with stabilizer and emulsifier properties in ice cream production. Tekin-Cakmak, Karasu [4] reported that the cold-pressed coconut oil by-products can be used in many food applications, especially low-fat emulsions due to high protein and carbohydrate contents. There are studies in the literature using different raw materials based on the production of plant-based drink and various dairy products [8,9,10]. However, no study was found on low-fat vegan ice cream using the cold-pressed coconut oil by-products.

The main purpose of this study is to produce a plant-based drink and ice cream using the by-products of cold-pressed coconut oil. In this study, firstly, a plant-based drink was produced from cold-pressed coconut oil by-products (COB drink) and compared with a commercial coconut drink. In the second stage, full-fat and low-fat plant-based ice cream samples using the COB drink were produced and compared to control ice cream samples (produced by a commercial coconut drink) in terms of rheological, sensorial, and thermal properties. Therefore, it was evaluated whether low-fat and full-fat ice cream could be produced from COB drink.

## 2. Materials and Methods

### 2.1. Material

COB was provided from ONEVA (Istanbul, Turkey). COB was obtained after mechanical screw press process. The coconut was ground up into coconut flakes and dried at 8% moisture content. Finally, screw press was applied to extract coconut oil and obtain COB. COB and distilled water were used to produce COB drink. COB drink, powdered sugar, sunflower oil, xanthan gum (as a stabilizer), and lecithin (as an emulsifier) were used for ice cream production. Coconut drink was bought from a local market as commercial control drink (Alpro Co., Wevelgem, Belgium). Xanthan gum and lecithin were obtained from Sigma-Aldrich (Sigma Chemical Co., St. Louis, MO, USA).

### 2.2. Preparation of Coconut By-Product Plant-Based Drink

**Figure 1:**
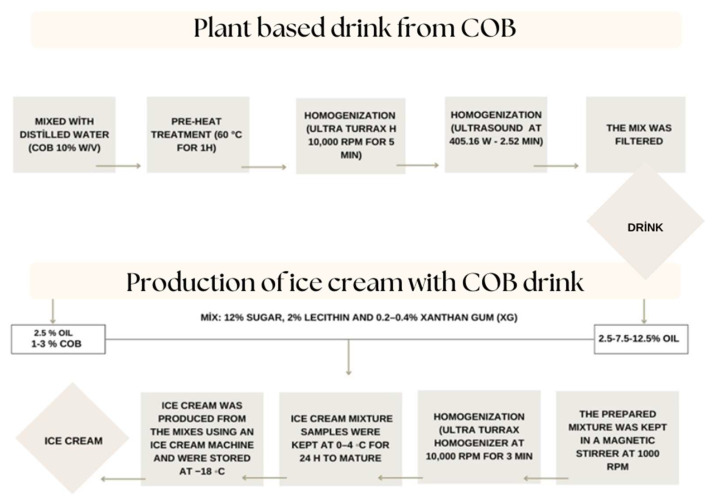
The flow sheet of production of coconut by-product drink and plant-based ice cream.

COB drink was produced by only drinking water and COB. COB was milled in a laboratory mill (PX-MFC 90 D, Kinematica, Malters, and Switzerland) and then sieved by No.140 mesh. Figure 1 indicated the flow sheet of the production of coconut by-product drink. The ground COB was blended with purified potable drinking water at 60 °C for 1 h to extract of protein and the other water soluble compounds by shaking water bath. The optimal concentration was determined as 10% (*w*/*v*) in our preliminary trial. The prepared mixture was homogenized with a homogenizer (Ultra Turrax, DAIHAN, HG-15D, Gang-Won-Do, Korea) at 10,000 rpm for 5 min. The optimum parameters of ultrasound homogenization were determined as 405.16 W and 2.52 min to decrease in particle size of plant-based drink. After the homogenization, the drink was filtered and the temperature of the drink was decreased to 10 °C and stored at 4 °C in the refrigerator. 

### 2.3. Preparation of Vegan Ice Cream

Figure 1 also indicates the flow sheet of the production of plant-based ice cream in lab scale. Three different ice cream samples as control samples (C1, C2, and C3) with different oil ratios (2.5, 7.5, and 12.5%) were produced with commercial coconut drink. Another control ice cream sample (COB-C3) was produced by using COB drink (at a 12.5% oil ratio). The low-fat ice cream samples (COB-1, COB-2, and COB-3) with a 25% oil ratio were stabilized by COB with a ratio of 1, 2, and 3%. 

Formulation of plant-based ice cream samples consisted of 2.5–12.5% oil, 12% sugar, 2% lecithin, and 0.35% xanthan gum (XG). To begin, XG and COB drink were weighed in accordance with the formulae established at the first stage of ice cream manufacturing. XG was gradually poured to completely dissolve it and held at 1000 rpm in a magnetic stirrer at 80 °C. Then, COB (1–3%) was added, blended, and weighted according to the low-fat ice cream recipe (COB-1, COB-2, and COB-3). Then, sugar was added sequentially. The lecithin and oil were mixed and added to all ice cream formulations. Then, the ice cream mixes were homogenized with a homogenizer (Ultra Turrax, DAIHAN, HG-15D, Gang-Won-Do, South Korea) at 10,000 rpm for 3 min. Following this procedure, all ice cream mixtures were kept at 0–4 °C for two days to mature. The mixtures were then used to make ice cream with an ice cream machine (DeLonghi IL Gelataio ICK5000, Treviso, Italy). Ice cream samples were stored at −18 °C for analysis.

### 2.4. Analysis of COB and Plant Drink Samples

#### 2.4.1. Physicochemical Analysis of COB

The dry matter content of COB was determined after the drying of samples at 105 °C for 2.5 h in an oven (FN 120, Nuve, Ankara, Turkey). Ash content of COB was determined at 550 °C for 6 h. The oil contents of COB were determined according to the Soxhlet method using hexane as a solvent. The protein content of COB was assessed by an automated nitrogen analyzer (FP 528, Leco, St. Joseph, MI, USA) using the Dumas method and calculated by multiplying the obtained nitrogen value (N:6.25). The pH value of the sample was determined at 25 °C using a pH meter (WTW- inoLab, Weilheim, Germany) in a suspension of 50% (*w*/*v*) COB in distilled water. The browning index (BI) value of COB was calculated according to the difference between the absorbance values of COB measured at 420 and 550 nm and expressed as optical density/g dry solid. Water activity (aw) is carried out with a water activity analyzer (Novasina Labtouch-Aw Water Activity Meter) at 30 °C.

#### 2.4.2. Bioactive Properties of COB

Total phenolic content (TPC) was performed by Folin–Ciocalteu method identified by Singleton and Rossi (1965) [11]. The TPC values were expressed as mg gallic acid equivalents (GAE)/g dry matter. The antioxidant activity values were determined using DPPH free radical according to the method identified by Singh et al. [12] and expressed as mg Trolox equivalents (TE)/g dry matter. 

#### 2.4.3. Physicochemical Analysis of Plant Drink Samples

The oil contents of the plant drink samples were determined according to the Gerber method. The protein content of the drink samples was assessed by the Kjeldahl method (N x 6.38). The pH of the drink sample was measured with a pH meter (WTW—inoLab, Weilheim, Germany).

#### 2.4.4. Particle Size and Zeta Potential Value of Drink Samples

The fat globule size distributions and zeta potential values were measured in the drink samples using Malvern Nanosizer (Malvern Instruments, Worcester, UK). The samples were diluted to 500 folds with distilled water to prevent multiple scattering effects and were homogenized by an ultrasonic water bath for 1 min [7]. 

#### 2.4.5. Color Parameters of Plant Drink Samples

The color parameters (L*: from 0 = black to 100 = white, a*: red (when +), gray (0), and green (−), and b*: yellow (+), gray (0), and blue (−)) of the plant drink samples were measured by a colorimeter (Konica Minolta, CR-400, Mississauga, ON, Canada) in the CIELAB system, and the measurements were carried out in triplicate at 25 °C [7]. 

#### 2.4.6. The Colloidal Stability of Plant Drink Samples

The colloidal stability of the plant drink samples was determined by phase separation when stored at 4 °C [13]. COB drink samples were transferred to 50 mL tubes, and the heights of the separated phases were measured on the 7th day. Colloidal stability was given as the sedimentation index, which was determined as follows:

Sedimentation (%) = Vn/V0 × 100,(1)

where Vn is the sedimentation volume at storage time (mL) and V0 is the initial volume (mL).

### 2.5. Analysis of Ice Cream Mixes

#### 2.5.1. Rheological Analyses

The rheological measurements (steady shear, dynamic rheological, and 3-ITT) of plant-based ice cream mixes were carried out by a stress-controlled rheometer (MCR 302, Anton Paar, North Ryde, Australia) equipped with parallel-plate configuration to shear and Peltier heating/cooling system. The probe was selected as PP50 (diameter 50 mm, Anton Paar, North Ryde, Australia), and the gap size was arranged as 0.5 mm. Each rheological measurement was performed three times at 25 °C.

The steady shear rheological properties of ice cream mixes were assessed at the shear rate between 0.1 s^−1^ and 100 s^−1^. The steady shear rheological data were fitted to the model parameters and were calculated using the Power law model and nonlinear regression.

(2) $$\tau =K\;\times \;\gamma^{n}\;$$

where the *τ* value is the shear stress (Pa), *K* is the consistency coefficient (Pas^n^), *γ* is the shear rate (s^−1^), and *n* is the flow behavior index. 

The dynamic rheological analysis of ice cream mixes was performed with parallel plate configuration. The linear viscoelastic region was first identified using the amplitude sweep test, which was conducted at 0.1% and 100% strain. Based on the results, the frequency sweep test was performed at the 0.1–10 Hz frequency range and 0.1–64 s^−1^ angular velocity (*ω*). The storage modulus (G′) and loss modulus (G′′) were determined depending on the angular velocity of the samples. The power law model and nonlinear regression were used to calculate the dynamic rheological parameters.

(3) $$G^{\prime }=K^{\prime }{\left(\omega \right)}^{n\prime \prime }$$

(4) $$G\prime \prime =K\prime \prime {\left(\omega \right)}^{n\prime \prime }$$

where the G′ value is storage modulus (Pa), G″ value is loss modulus (Pa), *ω* is angular velocity value (s^−1^), K′, K″ are consistency coefficient values (Pas^n^) and n′, n″ are flow behavior index values.

The 3-ITT rheological characteristics of ice cream samples were calculated as 150 s^−1^ for variable shear rate and 0.5 s^−1^ for constant shear rate. In determining the values, the linear viscoelastic region was taken into account, and in the samples, the linear viscoelastic region stops at 50 s^−1^. During the first 100 s of the first interval, the ice cream mixes were treated to a relatively low shear rate (0.5 s^−1^). The determined shear rate was applied to 150 s^−1^ for 40 s during the 2nd time interval. By exposing the samples to the low shear rate in the 1st time interval, the 3rd time interval investigated the dynamic rheological behavior of the ice cream mixes in the second time period. There was a change in the viscoelastic solid structure (G′) of the samples. A second-order structural kinetic model was used to simulate the behavior of samples in the 3rd time period.

(5) $${(\frac{G\prime -G_{e}}{G_{0}-G_{e}})}^{1-n}\;=\;\left(n-1\right)k\;\times \;t-1$$

where the G′ value represents the change in the storage module (Pa); G_0_ is the initial storage module value (Pa) in the 3rd time interval; G_e_ is the storage module at the moment when the product fully recovered, in other words, the storage module (Pa) at the moment when the product is fully balanced; and k is the thixotropic velocity constant.

#### 2.5.2. The Fat Globule Size Distributions and Zeta Potential Values of Ice Cream Mixes

The fat globule size distributions and zeta potential values were measured in the ice cream mixes using Malvern Nanosizer (Nanosizer, Malvern Instruments, Worcester, UK) and performed, as mentioned in Section 2.4.4 [14].

### 2.6. Analysis of the Ice Cream

#### 2.6.1. Color

The color parameters of the ice cream samples were calibrated with the CR-400 Chroma Meter, Konica, Minolta, Japan color measuring device, and the measurements were carried out in parallel from three different points [7]. L* value indicates brightness (0–100), a* value indicates color change value from red (+) to green (−), and b* value indicates a color change from yellow (+) to blue (−). Standard deviations of L*, a*, b* values of ice creams are given.

#### 2.6.2. Overrun

In order to determine the volume increase in the ice creams, ice cream samples were placed in the measuring cylinder, which was tared in such a way that there was no space up to a certain volume. The same process was applied to each ice cream and weighed on a precision scale. Afterward, the ice creams were allowed to melt at room temperature. Melted ice creams were filled to the same volume in the same cylinder measuring cup. Afterward, the melted ice creams were weighed with precision scales. The equation used in the calculation of the volume increase in ice creams is indicated.

#### 2.6.3. Thermal Properties of Ice Cream (DSC Analysis)

The thermal properties of plant-based ice cream samples were measured using a differential scanning calorimeter (DSC) by A DTA-DSC operating at atmospheric pressure (STA44gf3, Netzsch, Germany) [15]. Ice cream samples weighing 10 mg were put in a pre-weighed aluminum sample pan, sealed with a Quick Press pan crimper (T_zero_), and temperature data were collected from −20 to +50 °C in a nitrogen atmosphere at a heating rate of 1 °C/min. As a reference, an empty pan was utilized. The nitrogen gas flow rates for cooling were 50 mL/min. The onset temperatures (T_0_), T_f_, and ΔH_f_ of the ice formation and melting transitions were determined. The onset temperatures are defined as the point at which the tangent and baseline connect to the left of the melting peak. The steepest slope’s temperature was used to calculate freezing points. Extrapolating the baseline under the peak by linking the flat baseline before and after the melting peak and integrating the peak over the baseline yielded the enthalpy of fusion.

#### 2.6.4. Sensory Analysis

The sensory analysis was analyzed by 10 trained panelists, 5 of whom were male and 5 were female. After the panelists were informed about the product, fifty grams of ice cream samples (n = 7) were prepared for sensory evaluation. A table was used as a sensory evaluation form. The taste criteria of the ice cream samples were determined, such as aroma, cream taste, aftertaste, gummy structure, icy structure, roughness, foreign taste, color, melting in the mouth, and general acceptability. In sensory evaluations, they were asked to score on a scale ranging from 1 to 9 points. It was evaluated with the Hedonic scale of liking ((1): dislike extremely, (2): dislike very much, (3): dislike moderately, (4): dislike slightly, (5): neither like or dislike, (6): like slightly, (7): like moderately, (8): like very much, and (9): like extremely).

#### 2.6.5. Statistical Analysis

The statistical analysis was carried out using the Statistica software program (StatSoft, Inc., Tulsa, OK, USA). All the analyses were carried out in triplicate. The standard deviation and mean value were presented in all tables. ANOVA was conducted to determine the differences in all parameters of ice cream samples. Duncan’s multiple comparison tests at 95% significance level was used to determine the effect of oil and COB concentration on ice cream characteristics. 

## 3. Results and Discussion

### 3.1. Properties of COB and Plant Drink Samples

Table 1 showed the physicochemical and bioactive properties of the cold-pressed coconut oil by-product. The protein, fat, and carbohydrate content of COB was determined as 17.2%, 11.89%, and 60.07%, respectively. These results show that COB is rich in carbohydrates and is a considerable protein source. The pH, BI, and aw values were determined as 5.84, 1.77, and 0.417, respectively. The TPC and CUPRAC values of COB were 104.48 mg/100 g GAE and 106.58 mg/100 g TE. The results of TPC and antioxidant value in terms of CUPRAC indicated the importance of the bioactive property of COB. Some physicochemical properties of the commercial coconut drink and COB drink were also shown in Table 1. 

The fat, protein, and zeta potential values of COB drink were higher than those of the commercial coconut drink (CC drink). On the other hand, the pH and L* values of the CC drink were higher than the COB drink. The difference in pH values of drink samples can be attributed to the chemical structures and acid compounds of the sources from which they are obtained. The difference in L* value was probably due to the dark pigments naturally found in the structure of the raw materials of the drink and/or formed during the homogenization process. In addition, the particle size value of the COB drink was found to be lower than the commercial drink, indicating that an effective homogenization process was applied during COB drink production. The physical stabilities of the drink samples were 98% and 99% for COB drink and commercial drink, respectively. These results could be an indicator of the successful production of plant drink from COB.

### 3.2. Rheological Properties and Zeta Potential of Ice Cream Mixes 

#### 3.2.1. Steady Shear Rheological Properties of Ice Cream Mixes 

**Figure 2:**
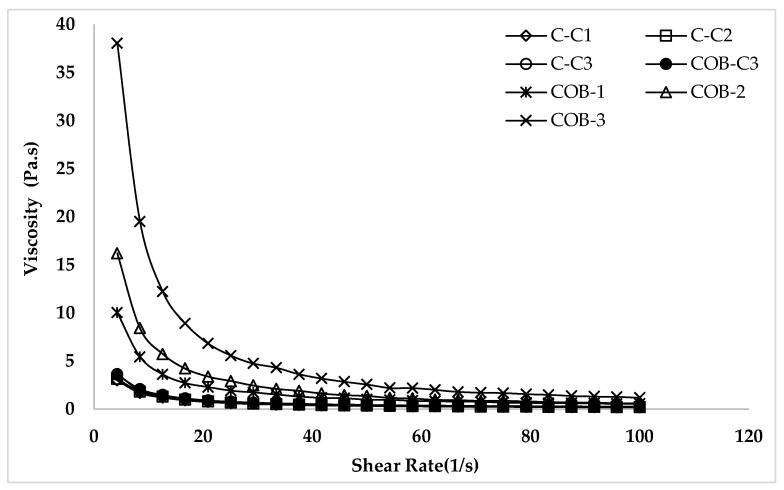
The steady shear rheological properties of ice cream mixes.

The effect on the flow curves of the plant-based ice cream mixes was assessed using the data of the steady shear rheological characteristics (Figure 2). For this purpose, control samples obtained from the commercial coconut drink with different fat content (2.5, 7.5%, and 12.5%) and the full-fat COB drink (12.5%) were compared with low-fat ice cream samples produced by the COB drink and stabilized with COB (1–3%). According to Figure 2, all samples displayed shear-thinning (pseudoplastic) behavior as the viscosity was reduced by increasing the shear rate. The results of our study were consistent with previous studies [16,17,18]. The structural breakdown of the intermolecular connection might explain the reduction in viscosity. Atik, Tekin Cakmak [7] determined that the low-fat ice cream samples stabilized with cold-pressed chia seed oil by-products showed shear-thinning properties. Additionally, Bekiroglu, Goktas [19] reported that plant-based ice cream samples prepared with walnut milk had a shear-thinning characteristic. The breaking influence of applied shear on aggregated components has been associated with the increased alignment of individual components in the structure of ice cream mixtures, which has been associated with the complex engagement of slightly destroyed micellar casein at the droplet surface in the ice cream mixes [20]. 

Table 2 showed that the power law model adequately described the flow behavior properties for all ice cream mixes (R2 values: 0.89–0.92). The flow behavior index (n) values varied from 0.14 to 0.19 were lower than 1 for all the ice cream mixes, indicating a non-Newtonian shear-thinning (pseudoplastic) behavior (Table 2). The shear-thinning (pseudoplastic) behavior demonstrates the high stability of the system properties under reduced shear rate processing settings, as well as the easy pumping of mix and the required texture and mouthfeel of the end product [10,11,12,13,14,15,16,17,18,19,20,21,22]. The consistency coefficient (K) values were 9.05–17.69 Pas^n^. As can be observed, the K value of the samples increased significantly as the oil and COB ratio increased, indicating a more viscous nature as a result of increased apparent viscosity in ice cream mixes. C1 had the lowest K value (9.05 Pas^n^), whereas the COB-3 exhibited the highest K value (17.69 Pas^n^). The oil/fat ratio is one of the vital factors affecting the consistency of emulsion products, such as ice cream mix [4]. In low-fat products, the decrease in consistency with the decrease in the oil content value should be compensated. The low-fat COB ice cream stabilized with COB showed higher K than those of all control samples. The increase in the consistency value with the increase in the COB ratio suggested that COB could be used in the structural improvement of plant-based ice cream.

The zeta potential values were utilized to estimate the stability of the emulsions by measuring the level of electrical repulsion force between particles. A high zeta potential suggests strong stability, whereas a low zeta potential indicates low stability because flocculation and close contact are prevented by a low repulsive force between the droplets [23]. Therefore, zeta potential values of the ice cream mixes varied from −38.77 mV to −41.47 mV, indicating that the ice cream mixes had high emulsion stability. Additionally, Atik, Tekin Cakmak [7] showed that the use of cold-pressed chia oil by-products as a stabilizer in low-fat ice cream samples can have a positive effect on zeta potential values and emulsion stability in ice cream. Table 2 also indicated that the particle sizes (d_32_) of ice cream mixes were determined between 1502.26 µm and 4.629.67 µm. These results indicated that the use of COB could have a positive effect on the fat droplet size and zeta potential values.

#### 3.2.2. Dynamic Rheological Properties of Ice Cream Mixes

**Figure 3:**
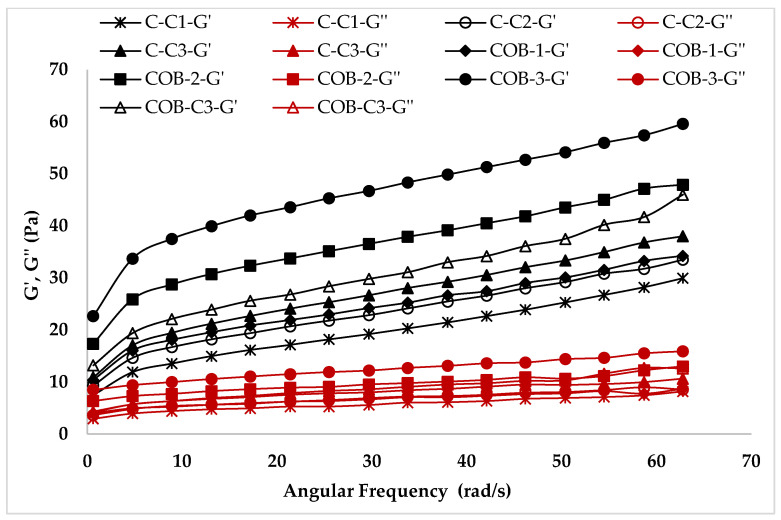
The dynamic rheological properties of ice cream mixes.

The dynamic rheological properties of ice cream mixes were investigated to evaluate the solid or liquid nature of ice cream samples. The frequency sweep test can simulate the solid/liquid behavior of ice cream samples while being chewed in the mouth [24], which enables a thorough assessment of the effect of oil and COB concentration on ice cream quality. The evidence of gel-like behavior in ice cream samples is the increase in G′ and G″ values of samples with increasing frequency [25]. As can be observed in Figure 3, the value of G′ for all samples was higher than the value of G′′ in all frequency ranges, suggesting that the solid-like behavior of all plant-based ice cream samples predominates over the liquid character. The viscoelastic solid character is desired rheolocial properties expected from ice cream [26]. The frequency data indicated that all plant-based samples, including low-fat ice cream, could show standard ice cream characteristics. 

Table 3 presents K′, K″, n′, and n″ values computed using the power law model. The model’s R^2^ values were confirmed to be more than 0.95. As shown in Table 3, the samples’ K′ and K″ values were in the ranges of 5.76–26.59 and 2.48–7.70, respectively, while the n′ and n″ values were in the ranges of 0.33–0.38 and 0.18–0.26, respectively. The K′ values were lower than the K″ values of the plant-based ice cream mixes, indicating that the mixes were viscous. Atalar, Kurt [27] reported a similar result for ice cream samples enhanced with protein-rich hazelnut drink. The COB-3 sample has the greatest K′ and K″ values, whereas C1 has the lowest. The greater K value of COB stabilized samples can be attributed to synergistic interactions between COB and other ice cream components, which can result in the improvement of food quality and extended culinary applications thanks to the enhancement of functional qualities. The solid character grows as the K′ values increase with the COB content, whilst lower n′ values suggest increased network stability [28].

#### 3.2.3. Thixotropic Behavior of Ice Cream Mixes

**Figure 4:**
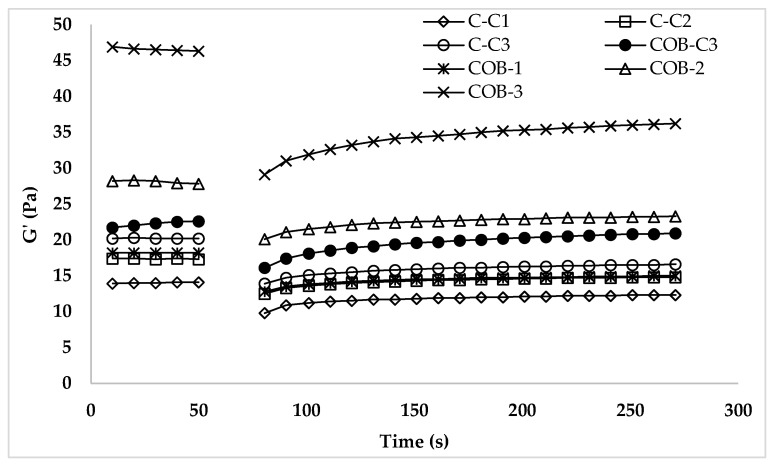
The 3-ITT rheological properties of ice cream mixes.

The three interval thixotropy test (3-ITT) simulates the sudden shear stress/shear rate deformation of ice cream mixes and gives information about the structural recovery of ice cream mixes following abrupt deformation. Figure 4 showed that all ice cream mixes in the 3rd interval indicated that the thixotropic behavior was only based on G′ values due to its dominant characteristic for the ice cream mixes. As shown in Figure 4, the structural recovery tendency of all ice cream mixes changed by the oil and COB ratio. The low-fat ice cream mix demonstrated the lowest structural recovery while COB-3 indicated the highest recovery. The lowest structural recovery can be explained by the fact that ice cream cannot be quickly restored to its original structure after deformation caused by homogenization or pumping during processing because of the high viscosity and strength of structural molecular interactions [29,30]. According to Atik, Tekin Cakmak [7], the by-products of cold-pressed chia oil enhanced the thixotropic behavior of ice cream samples following abrupt deformation. In the third period, our ice cream samples revealed recoverable attributes that are consistent with earlier research by Atik, Tekin Cakmak [7]. The results of this study show that all plant-based ice cream mixes exhibit the recovery tendency expected from conventional ice cream mixes.

The second-order structural kinetic model’s parameters (G_0_, G_e_, k, G_e_/G_0_) are shown in Table 4. The tendency to more quickly recover increases with the number of G_e_/G_0_. The greatest G_0_, G_e_, G_e_/G_0_, and k values were found in samples C1 and COB-3, indicating that sample COB-3 exhibited the most thixotropic behavior. The COB showed similar recovery characteristics with the high-oil control sample. The tendency to recover is greater the higher the thixotropic rate constant value signifies samples. These findings suggested that COB may be used to enhance the ice cream mix’s recoverable attributes.

### 3.3. Analysis of the Ice Cream

#### 3.3.1. Color

Consumer taste is defined by certain visual and taste criteria. As a result, color is a significant aspect of the first impression when consumers accept or reject a food product based on their sensory expectations. Color parameters (L*, a*, b*) of ice cream samples were presented in Table 5. As seen in Table 5, L*, a*, and b* values were 69.08–78.65, 0.05–0.47, and 7.48–11.04, respectively. The highest L* value of the ice cream samples was observed in the C1 (78.65) and the lowest L* value in the C3 sample (69.08). The L* values of the samples prepared with COB were lower than the control samples and were statistically different (*p* < 0.05). The highest a* value of the ice cream samples was observed with 0.32 in C3 and the lowest a* value of the C1 was observed. The a* values of ice cream samples below zero indicate that green color is more dominant than red in these samples. The effect of the oil and COB content used on the a* values of the samples were found to be significant (*p* < 0.05). Whereas the highest b* value was observed in COB 3, the lowest b* value was observed in the C3 sample and the b* values of the samples were found to be statistically significant (*p* < 0.05). It is thought that the difference between the color values of the samples, especially the L*, a*, and b* values, is due to the amount of oil and COB. When Ürkek [31] examined the color values of ice cream samples containing salep and chai powder in different ratios, L*, a*, and b* values were found to be 76.18–86.84, (−3.64)–(−2.71), and 8.58–12.10, respectively.

#### 3.3.2. Overrun

The overrun values of plant-based ice cream samples were between 22.02 and 28.33% (Table 6). Statistical research demonstrated that the amount of COB and additional oil had a significant effect (*p* < 0.05) on the overrun values. The sample of the COB-C3 and COB3 containing 3% of COB and 2.5% oil showed a higher overrun value than C2 (7.5% oil). The increase in overrun was supplied so that it increases as the oil content increases and it increases further with the increase in the COB amount. Tekin et al. (2021) [4] reported that COB contained 60.82% carbohydrate and 16.36% protein. The greater increase in volume value with the addition of COB in ice cream samples compared to the addition of fat may be attributed to the protein and carbohydrate in the COB content. Additionally, proteins can enhance overrun values because of their emulsification capabilities, which help to increase foam stability [32].

Overrun is related to the quantity of air introduced into the ice cream manufacturing process [33]. Chang and Hartel [34] reported that the formation of air cells can be affected by the fat, emulsifier, and stabilizer contents, as well as the processing variables (whipping temperature and freezing power). Therefore, the utilization of carbohydrate-based fat substitutes, which have a viscous property that may decrease whipping capacity, is most likely responsible for the low overrun values of orange fiber ice creams. COB improved not only carbohydrates but also protein content and overrun properties. However, it can be concluded that with the addition of 3% COB, ice cream with an overrun value can be obtained, albeit not as much as full-fat ice cream (C3), but as medium-fat ice cream (C2).

#### 3.3.3. Thermal Properties of the Ice Cream

Table 6 indicated that the thermal properties (T_onset_, T_end_, T_f_, ΔT, and ΔH_f_,) associated with ice crystal melting of ice cream samples were determined by DSC. T_onset_, T_end_, and T_f_ were between −13.66 and −61.11 °C, 4.45 and 5.26 °C, and −4.60 and −3.31 °C, respectively. Melting resistance is the capacity of ice cream to withstand melting when subjected to high temperatures. The temperature triggers the creation of an endothermic peak provided by the DSC’s heating system. The amount of energy leaving the system is represented by the melting enthalpy (ΔH_f_), which is determined by integrating with the endothermic peak’s area. ΔHf values of the ice cream samples ranged from 144.70 J/g to 172.70 J/g. Depending on whether the water in the ice cream is free or bound, ice creams with higher frozen water will have ice formation, resulting in a reduction in enthalpy. Ice creams with lower frozen water have less melting enthalpy [35]. Therefore, low-fat ice cream (C1, 2.5% oil) has the highest ΔH_f_ value (172.70 J/g). The increase in the liquid fraction with the decrease in the fat ratio may have caused an increase in the amount of frozen water and thus an increase in the freezing enthalpy. The samples stabilized with COB contained less oil content but showed similar thermal properties to the oily sample. The ΔH_f_ value decreased with increasing COB content. This result can be explained by the water retention of the protein and polysaccharides in the COB and the decrease in the amount of freezable water. In general, the temperature range (ΔT) is a measure of the uniformity of ice crystal size distribution. Therefore, a narrow melting temperature range represents a more homogenous distribution of ice crystals melting at a lower temperature range [36]. ΔT were between 10.80 and 18.06 °C. The enrichment of ice cream COB improves the formation of small ice crystals thanks to polysaccharides and protein content, thus contributing to improving the perception of texture and increasing ice crystal stability during cold storage.

#### 3.3.4. Sensory

The sensory parameters of plant-based ice cream samples were presented in Table 7. As seen in Table 7, there were no significant differences between the sensory scores of ice cream samples. Full-fat ice cream samples (COB-C3 and C3) showed the highest scores for all sensory parameters. Full-fat and low-fat ice cream samples produced from the COB drink had similar properties in all sensory parameters. COB-3% (low-fat ice cream), on the other hand, indicated the similar sensory properties of the full-fat sample (COB-C3 and C3), except for the cream taste. A change in the color parameter is expected compared to the control samples (C1, C2, C3, and COB-C3) with the addition of COB.

There was no significant difference in the general acceptability between the COB-containing sample (COB 1–2–3) and control samples (C1, C2, C3, and COB-C3). This demonstrated that the color difference noticed in the low-fat ice cream samples with the addition of full-fat COB-C3 and COB had no negative impact on the ice cream’s consumability. As the consistency increases in ice cream samples, the melting resistance increases. Oil content is very important for ice cream samples because the quantity of fat aggregates was substantially linked with ice cream melting resistance. Melting resistance was found in the ice cream with the highest melting resistance. Similar to our study, Atik, Tekin Cakmak [7] revealed that the inclusion of fat replacements did not lead to unfavorable changes in ice cream sensory scores. These findings demonstrated that ice creams made from COB drink could be enhanced without compromising sensory features and that low-fat plant-based ice cream samples could be created in the same manner to obtain full-fat ice cream quality attributes.

## 4. Conclusions

The plant-based product consumption has increased in recent years thanks to ethical reasons, lactose intolerance, and diet preferences (vegan or vegetarian). However, plant-based drinks are more expensive compared to cow drink. Therefore, a significant option to lower the price of plant drink products may be found in the utilization of cold-pressed coconut oil by-products in the manufacturing of plant-based drinks. The oil industry may also benefit from gaining a valuable by-product identity. Based on its physicochemical and bioactive properties, COB can be preferred for successful plant drink production. The fat, protein, and zeta potential values of the COB drink were higher than those of the commercial coconut drink. All ice cream samples showed shear thinning (pseudoplastic), solid-like structure, and recoverable characters, which are expected rheological properties from conventional ice cream. In this study, it has been shown that ice cream samples produced from a COB drink can be developed without negatively impacting their sensory qualities, and sensory properties similar to full-fat ice cream can be obtained by using COB as a stabilizer in low-fat ice cream production. This study demonstrated that COB may be successfully employed in plant-based drink manufacturing and low-fat plant-based ice cream, providing a fresh viewpoint for the evaluation of this low-economic by-product.

## Figures and Tables

**Table 1:** Physicochemical properties of coconut oil by-products and plant drink samples.

Physicochemical and Bioactive Properties of Coconut Oil By-Products (COB)		
Physicochemical Properties	Dry Matter %	92.10 ± 0.04
	Ash %	2.94 ± 0.00
	Oil %	11.89 ± 0.01
	Protein %	17.20 ± 0.80
	Carbohydrate %	60.07 ± 1.44
	pH	5.84 ± 0.02
	BI	1.77 ± 0.01
	a_w_	0.417 ± 0.20
	TPC (mg/100 g)	104.49 ± 1.06
Bioactive Properties	CUPRAC (mgTE/100 g)	106.58 ± 10.38
	DPPH (mg TE/100 g)	3.01 ± 0.00
Physicochemical properties of plant drink		
	COB drink	CC drink
Oil	2.08 ± 0.01	1.90 ± 0.01
Protein	2.43 ± 0.02	1.05 ± 0.1
pH	5.91 ± 0.01	7.23 ± 0.01
Size (nm)	403.15 ± 0.04	1137.5 ± 0.44
PDI	0.65 ± 0.19	0.6 ± 0.08
Zeta Potential (mV)	−16.95 ± 0.21	−12.35 ± 0.21
L*	65.70 ± 0.58	72.00 ± 0.13
a*	−1.27 ± 0.39	−0.91 ± 0.04
b*	5.68 ± 0.58	3.67 ± 0.43
7th Day Stability (%)	98 ± 0.00	99 ± 0.00

COB: cold-pressed coconut oil by-product; COB drink: plant-based drink produced by coconut by-products; CC: commercial coconut drink.

**Table 2:** Steady rheological properties, zeta potential, and particle size of plant-based ice cream mixes.

Samples	Oil (%)	COB (%)	K (Pas^n^)	n	R^2^	Zeta Potential (mV)	Particle Size, d_32_ (µm)
COB-C3	12.5	-	10.23 ± 0.13 ^cd^	0.19 ± 0.00 ^a^	>0.95	−34.55 ± 0.03 ^a^	1502.26 ± 2.52 ^c^
C1	2.5	-	9.05 ±0.04 ^d^	0.14 ± 0.00 ^c^	>0.95	−40.07 ± 0.55 ^bc^	2539.33 ± 0.20 ^bc^
C2	7.5	-	9.67 ± 0.08 ^d^	0.15 ± 0.00 ^bc^	>0.95	−41.47 ± 0.58 ^c^	3582.00 ± 1.90 ^ab^
C3	12.5	-	11.14 ± 0.31 ^bc^	0.15 ± 0.00 ^bc^	>0.95	−39.73 ± 0.98 ^bc^	4.629.67 ± 0.26 ^a^
COB-1	2.5	1	9.29 ± 0.00 ^d^	0.16 ± 0.00 ^b^	>0.95	−38.80 ± 0.52 ^b^	1694.7 ± 0.62 ^c^
COB-2	2.5	2	12.22 ± 0.14 ^b^	0.15 ± 0.00 ^bc^	>0.95	−38.77 ± 0.81 ^bc^	2271.3 ± 0.62 ^c^
COB-3	2.5	3	17.69 ± 0.46 ^a^	0.14 ± 0.00 ^bc^	>0.95	−39.27 ± 1.72 ^bc^	1855.7 ± 0.46 ^c^

The different lowercase letters in the same columns indicate statistical differences. COB: Coconut oil by-product, C1–C3: Ice cream mixes produced with commercial coconut drink; COB-C3: Ice cream mix produced with drink obtained from coconut oil by-product; COB1–3: Low-fat ice cream mixes produced with drink obtained from coconut oil by-product stabilized with COB.

**Table 3:** Dynamical rheological properties of plant-based ice cream mixes.

Samples	K′	n′	R^2^	K″	n″	R^2^
COB-C3	10.28 ± 0.12 ^c^	0.33 ± 0.00 ^ab^	0.97 ± 0.00	3.20 ± 0.05 ^cd^	0.31 ± 0.00 ^a^	>0.95
C1	5.76 ± 0.09 ^e^	0.38 ± 0.01 ^a^	0.97 ± 0.00	2.48 ± 0.02 ^d^	0.26 ± 0.00 ^b^	>0.95
C2	7.85 ± 0.28 ^d^	0.33 ± 0.01 ^ab^	0.98 ± 0.00	3.24 ± 0.11 ^cd^	0.23 ± 0.01 ^bc^	>0.95
C3	9.47 ± 0.56 ^c^	0.32 ± 0.01 ^b^	0.98 ± 0.00	3.89 ± 0.13 ^c^	0.23 ± 0.01 ^bc^	>0.95
COB1	9.14 ± 0.26 ^cd^	0.30 ± 0.00 ^b^	0.98 ± 0.00	3.56 ± 0.13 ^c^	0.19 ± 0.01 ^cd^	>0.95
COB2	16.82 ± 0.30 ^b^	0.24 ± 0.00 ^c^	0.98 ± 0.00	5.42 ± 0.01 b	0.18 ± 0.00 ^d^	>0.95
COB3	26.59 ± 0.33 ^a^	0.22 ± 0.00 ^c^	0.99 ± 0.00	7.70 ± 0.39 ^a^	0.17 ± 0.02 ^d^	>0.95

The different lowercase letters in the same columns indicate statistical differences. COB: Coconut oil by-product, C1–C3: Ice cream mixes produced with commercial coconut drink; COB-C3: Ice cream mix produced with drink obtained from coconut oil by-product; COB1–3: Low-fat ice cream mixes produced with drink obtained from coconut oil by-product stabilized with COB.

**Table 4:** The 3-ITT rheological properties of plant-based ice cream mixes.

Samples	G_0_	G_e_	G_e_/G_0_	K × 1000	R^2^
COB-C3	14.44 ± 0.27 ^c^	20.91 ± 0.02 ^bc^	1.44 ^a^	3.51 ^a^	>0.99
C1	9.85 ± 0.71 ^e^	12.50 ± 0.01 ^d^	1.26 ^c^	3.16 ^e^	>0.99
C2	11.24 ± 0.25 ^de^	14.80 ± 0.72 ^cd^	1.31 ^c^	3.31 ^d^	>0.99
C3	13.82 ± 1.28 ^cd^	19.49 ± 0.46 ^bc^	1.41 ^a^	3.41 ^b^	>0.99
COB1	12.49 ± 0.71 ^cde^	15.64 ± 0.72 ^cd^	1.26 ^c^	3.12 ^e^	>0.99
COB2	18.88 ± 0.16 ^b^	25.28 ± 0.71 ^b^	1.33 ^bc^	3.30 ^d^	>0.99
COB3	25.01 ± 0.69 ^a^	33.87 ± 0.61 ^a^	1.35 ^b^	3.35 ^c^	>0.99

The different lowercase letters in the same columns indicate statistical differences. COB: Coconut oil by-product, C1–C3: Ice cream mixes produced with commercial coconut drink; COB-C3: Ice cream mix produced with drink obtained from coconut oil by-product; COB1–3: Low-fat ice cream mixes produced with drink obtained from coconut oil by-products stabilized with COB.

**Table 5:** Color values and sensory analysis of vegan ice cream mixes.

	L*	a*	b*
COB-C3	78.02 ± 0.08 ^a^	0.29 ± 0.04 ^b^	9.11 ± 0.07 ^bc^
C1	78.65 ± 0.12 ^a^	0.05 ± 0.00 ^d^	8.73 ± 0.11 ^c^
C2	71.02 ± 0.14 ^bc^	0.12 ± 0.01 ^c^	7.56 ± 0.30 ^de^
C3	69.08 ± 0.54 ^d^	0.32 ± 0.02 ^b^	7.48 ± 0.11 ^e^
COB1	71.45 ± 0.16 ^b^	0.47 ± 0.02 ^a^	8.22 ± 0.13 ^cd^
COB2	70.35 ± 0.17 ^c^	0.15 ± 0.04 ^c^	9.52 ± 0.11 ^b^
COB3	71.79 ± 0.14 ^b^	0.07 ± 0.01 ^d^	11.04 ± 0.33 ^a^

The different lowercase letters in the same columns indicate statistical differences. COB: Coconut oil by-product, C1–C3: Ice cream mixes produced with commercial coconut drink; COB-C3: Ice cream mix produced with drink obtained from coconut oil by-product; COB1–3: Low-fat ice cream mixes produced with drink obtained from coconut oil by-product stabilized with COB.

**Table 6:** Thermal properties and overrun of the ice cream samples.

	T_0_ (°C)	T_end_ (°C)	T_f_ (°C)	ΔT (°C)	ΔH_f_ (J/g)	Overrun (%)
COB-C3	−8.41 ± 0.01 ^b^	5.26 ± 0.02 ^a^	−4.46 ± 0.01 ^c^	13.67 ± 0.02 ^c^	144.7 ± 0.52 ^e^	26.88 ± 0.03 ^b^
C1	−10.65 ± 0.01 ^c^	5.09 ± 0.01 ^ab^	−3.47 ± 0.01 ^ab^	15.74 ± 0.01 ^b^	172.70 ± 0.42 ^a^	23.75 ± 0.02 ^f^
C2	−13.60 ± 0.22 ^d^	4.45 ± 0.09 ^d^	−4.60 ± 0.03 ^c^	18.06 ± 0.32 ^a^	157.05 ± 0.21 ^c^	23.97 ± 0.01 ^e^
C3	−8.38 ± 0.05 ^b^	4.51 ± 0.06 ^cd^	−3.71 ± 0.16 ^b^	12.88 ± 0.01 ^d^	151.20 ± 0.28 ^d^	25.21 ± 0.04 ^c^
COB1	−6.11 ± 0.01 ^a^	4.70 ± 0.00 ^c^	−3.31 ± 0.01 ^a^	10.80 ± 0.01 ^e^	163.15 ± 0.21 ^b^	22.02 ± 0.02 ^g^
COB2	−8.60 ± 0.01 ^b^	5.01 ± 0.01 ^b^	−3.45 ± 0.01 ^a^	13.62 ± 0.01 ^c^	155.55 ± 0.78 ^c^	24.18 ± 0.04 ^d^
COB3	−8.63 ± 0.21 ^b^	5.02 ± 0.01 ^b^	−4.50 ± 0.00 ^c^	13.65 ± 0.22 ^c^	145.25 ± 0.21 ^e^	28.33 ± 0.01 ^a^

The different lowercase letters in the same columns indicate statistical differences. COB: Coconut oil by-product, C1–C3: Ice cream mixes produced with commercial coconut drink; COB-C3: Ice cream mix produced with drink obtained from coconut oil by-product; COB1–3: Low-fat ice cream mixes produced with drink obtained from coconut oil by-product stabilized with COB.

**Table 7:** Sensorial properties of the plant-based ice cream samples.

Samples	Color and Appearance	Icy Structure and Consistency	Foreign Taste and Smell	Cream Taste	Melting Resistance	General Acceptance
COB-C3	8.28 ± 0.26 ^a^	8.64 ± 0.47 ^a^	8.07 ± 0.53 ^a^	8.92 ± 0.53 ^a^	8.05 ± 1.15 ^a^	8.42 ± 0.97 ^a^
C1	8.42 ± 0.45 ^a^	8.02 ± 0.64 ^ab^	8.04 ± 0.18 ^a^	7.00 ± 0.48 ^a^	7.22 ± 0.55 ^a^	7.78 ± 0.39 ^a^
C2	8.64 ± 0.37 ^a^	7.35 ± 1.05 ^b^	8.55 ± 0.45 ^a^	7.65 ± 0.78 ^a^	7.53 ± 0.45 ^a^	8.02 ± 0.34 ^a^
C3	8.5 ± 0.57 ^a^	8.55 ± 0.35 ^a^	8.42 ± 0.78 ^a^	9.05 ± 0.53 ^a^	8.15 ± 0.37 ^a^	8.85 ± 0.89 ^a^
COB1	7.71 ± 0.89 ^a^	7.50 ± 0.77 ^ab^	7.64 ± 0.74 ^a^	6.35 ± 0.89 ^a^	7.15 ± 0.89 ^a^	8.35 ± 0.55 ^a^
COB2	8.00 ± 0.57 ^a^	8.07 ± 0.61 ^ab^	8.08 ± 0.99 ^a^	6.55 ± 1.15 ^a^	8.05 ± 0.89 ^a^	8.12 ± 0.44 ^a^
COB3	8.71 ± 0.56 ^a^	8.21 ± 0.39 ^ab^	8.64 ± 1.46 ^a^	6.92 ± 1.21 ^a^	8.50 ± 1.11 ^a^	8.57 ± 0.78 ^a^

The different lowercase letters in the same columns indicate statistical differences. COB: Coconut oil by-product, C1–C3: Ice cream mixes produced with commercial coconut drink; COB-C3: Ice cream mix produced with drink obtained from coconut oil by-product; COB1–3: Low-fat ice cream mixes produced with drink obtained from coconut oil by-product stabilized with COB.

## Data Availability

The authors declare that all the data supporting the findings of this study are available within the article.
